# Effects of inaccuracies in arterial path length measurement on differences in MRI and tonometry measured pulse wave velocity

**DOI:** 10.1186/s12872-017-0546-x

**Published:** 2017-05-10

**Authors:** Jonathan R Weir-McCall, Faisel Khan, Deirdre B Cassidy, Arsh Thakur, Jennifer Summersgill, Shona Z Matthew, Fiona Adams, Fiona Dove, Stephen J Gandy, Helen M Colhoun, Jill JF Belch, J Graeme Houston

**Affiliations:** 1 Division of Molecular and Clinical Medicine, Medical Research Institute, University of Dundee, Dundee, DD1 9SY UK; 2 NHS Tayside Medical Physics, Ninewells Hospital, Dundee, Scotland, UK; 3 Centre for Genomic and Experimental Medicine, The University of Edinburgh, Western General Hospital, Crewe Road, Edinburgh, EH4 2XU Scotland, UK

**Keywords:** Arteriosclerosis, MRI, PWV, Diabetes, Cardiovascular

## Abstract

**Background:**

Carotid-femoral pulse wave velocity (cf-PWV) and aortic PWV measured using MRI (MRI-PWV) show good correlation, but with a significant and consistent bias across studies. The aim of the current study was to evaluate whether the differences between cf.-PWV and MRI-PWV can be accounted for by inaccuracies of currently used distance measurements.

**Methods:**

One hundred fourteen study participants were recruited into one of 4 groups: Type 2 diabetes melltus (T2DM) with cardiovascular disease (CVD) (*n* = 23), T2DM without CVD (*n* = 41), CVD without T2DM (*n* = 25) and a control group (*n* = 25). All participants underwent cf.-PWV, cardiac MRI and whole body MR angiography(WB-MRA). 90 study participants also underwent aortic PWV using MRI. cf.-PWV_EXT_ was performed using a SphygmoCor device (Atcor Medical, West Ryde, Australia). The true intra-arterial pathlength was measured using the WB-MRA and then used to recalculate the cf.-PWV_EXT_ to give a cf.-PWV_MRA_.

**Results:**

Distance measurements were significantly lower on WB-MRA than on external tape measure (mean diff = −85.4 ± 54.0 mm,*p* < 0.001). MRI-PWV was significantly lower than cf.-PWV_EXT_ (MRI-PWV = 8.1 ± 2.9 vs. cf.-PWV_EXT_ = 10.9 ± 2.7 ms^−1^,*p* < 0.001). When cf.-PWV was recalculated using the inter-arterial distance from WB-MRA, this difference was significantly reduced but not lost (MRI-PWV = 8.1 ± 2.9 ms^−1^ vs. cf.-PWV_MRA_ 9.1 ± 2.1 ms^−1^, mean diff = −0.96 ± 2.52 ms^−1^,*p* = 0.001). Recalculation of the PWV increased correlation with age and pulse pressure.

**Conclusion:**

Differences in cf.-PWV and MRI PWV can be predominantly but not entirely explained by inaccuracies introduced by the use of simple surface measurements to represent the convoluted arterial path between the carotid and femoral arteries.

## Background

Arteriosclerosis is the process of arterial stiffening that has significant pathophysiological implications and is strongly associated with cardiovascular events [1]**.** It is predominantly a product of age and pulse pressure, reflecting the effects of repetitive strain on the elastic fibers of the arterial wall [2]. Pulse wave velocity is a well established marker of arterial stiffening with important prognostic implications, and as a result of such has now been incorporated into management guidelines for the treatment of hypertension [3]. Carotid femoral pulse wave velocity (cf-PWV) is the most commonly used technique for the measurement of PWV, however it relies on the use of surface measures of distance to represent the convoluted path of the underlying arterial tree, introducing potential errors in PWV calculation.

Use of Magnetic Resonance Imaging (MRI) for the measurement of PWV (MRI-PWV) has become increasingly prominent due to its ability to measure directly the central aortic PWV coupled to recent advances in the technique allowing high temporal resolution imaging and reduced image acquisition time [4]. The two techniques correlate well with each other, but with a significant and consistent bias between the two techniques across studies with cf.-PWV consistently being approximately 1.6–1.7 ms^−1^ higher than MRI-PWV [5, 6]. This is consistent with observations of 1.9 ms^−1^ difference between the aortic pulse wave velocity measured invasively and the cf-PWV, however this previous study also used a direct carotid-femoral measurement which is known to overestimate distance travelled and thus PWV by approximately 25% [7]. Previous work has demonstrated both a clear discrepancy in the distances measured using a tape measure and the actual arterial pathlength travelled by the pulse wave due to the effects of age and obesity confounding both arterial length and body contour measurements [8]. However the muscular and elastic properties of the aorta and its branch vessels is also known to change throughout their length which in turn affects their stiffness and by extension PWV [6, 9].

It is thus not currently clear whether the differences in PWV between the two techniques are due to inaccuracies in distance measurement in cf-PWV, the lower temporal resolution of MRI-PWV or the changing elastic properties throughout the arterial tree. The effects of this bias are also not clear. The aim of the current study was therefore to evaluate whether the differences between cf.-PWV and MRI-PWV in a mixed cohort of patients with and without diabetes mellitus and symptomatic cardiovascular disease can be accounted for by inaccuracies of currently used distance measurements and whether recalculation of cf.-PWV using accurate distance measurements results in improved detection of cardiovascular disease.

## Methods

### Participants

The study was a single centre observational sub-study of the multicentre SUMMIT (SUrrogate markers for Micro- and Macrovascular hard endpoints for Innovative diabetes Tools) study which was designed to analyse cardiovascular biomarkers in diabetes. Recruitment criteria, strategy and study protocol have been described in detail previously [10, 11], but in summary, subjects were recruited and categorised into 4 groups based on their history of type 2 diabetes and cardiovascular disease (CVD) as follows: *Group 1*: Type 2 diabetes mellitus (T2DM) with a prior clinical diagnosis of cardiovascular disease that included coronary artery disease (CAD), cerebrovascular disease and/or lower extremity arterial disease (LEAD); *Group 2*: Type 2 diabetes mellitus with no clinical evidence of cardiovascular disease; *Group 3*: Absence of diabetes mellitus with clinical evidence of CAD, cerebrovascular disease and/or LEAD; *Group 4*: Healthy controls, with no biochemical evidence of diabetes mellitus (see below) and no clinical evidence of cardiovascular disease. All participants had a detailed clinical history and examination performed, bloods taken for renal function, cholesterol and HbA1c, and underwent whole body magnetic resonance angiography (MRA), cardiac magnetic resonance imaging (CMR) and cf.-PWV.

### Carotid-femoral pulse wave velocity

Carotid-femoral PWV was measured using a SphygmoCor device (Atcor Medical, West Ryde, Australia). A blood pressure (BP) cuff was attached to the left arm, and three electrocardiogram (ECG) electrodes (lead I) were attached. External distance measurements were performed using a tape measure, and a proximal (carotid to sternal notch) and distal (sternal notch to umbilicus and umbilicus to femoral) measure with final distance used for calculation being the proximal distance subtracted from the distal distance. After resting for 5 min, the BP was measured three times at one-minute intervals, and the mean value of the two final measurements was recorded. The carotid and femoral pulses were captured using the SphygmoCor device with cf.-PWV automatically calculated as the measured distance divided by the differences in time between the R wave of the ECG and the foot of the carotid and femoral pulse curves. For the remainder of the paper, cf.-PWV calculated using the external tape measure distance shall be referred to as cf.-PWV_EXT_.

### Magnetic resonance imaging

Images were acquired on a 32 RF receiver channel, 3 Tesla MRI scanner (Magnetom Trio, Siemens, Erlangen, Germany). For whole body coverage, a combination of six RF coils including head matrix, neck matrix, spine matrix, two body matrix and peripheral angiography phased array RF surface coils were used. Subjects where placed supine, head first into the magnet bore. The imaging protocol was carried out in 3 phases: i) MRA of the thoracic and neck, and distal lower limbs, ii) CMR including late gadolinium enhancement (LGE) and iii) MRA of the abdomen, pelvis and proximal lower limb.

#### Whole body magnetic resonance angiography protocol

Whole body magnetic resonance angiography (WB-MRA) was performed using 4 overlapping data sets covering: head, neck and thorax (station 1), abdomen and pelvis (station 2), upper legs (stations 3) and lower legs (station 4). All stations were acquired using a coronal spoiled FLASH (Fast Low Angle Shot) sequence (repetition time TR = 2.6–3.47 ms; echo time TE = 0.96–1.21 ms; flip angle = 16–37°; pixel area = 1.1–1.5 mm^2^ and slice thickness = 1–1.4 mm, slight variation according to station and participant body habitus). [12].

First, station 1 and 4 pre-contrast images were acquired. Following this an injection of 10 ml of 0.5 M Gadoterate meglumine (Guerbet, Villipinte, France) followed by a 20 ml saline flush were administered at a rate of 1.5 ml/s. Acquisition of station 1 was triggered when the contrast reached the aortic arch (timed using a fluoro sequence through the aortic arch), following which three sequential acquisitions of station 4 were performed. Cardiac MRI was performed after this first injection (see next section for details) before stations 2 and 3 image acquisition began. After the cardiac acquisition was completed, pre contrast images were acquired of both stations following which post-contrast images were acquired after an injection of 15 ml gadoterate meglumine and 20 ml saline flush, both administered at a rate of 1.5 ml/s.

**Fig. 1:**
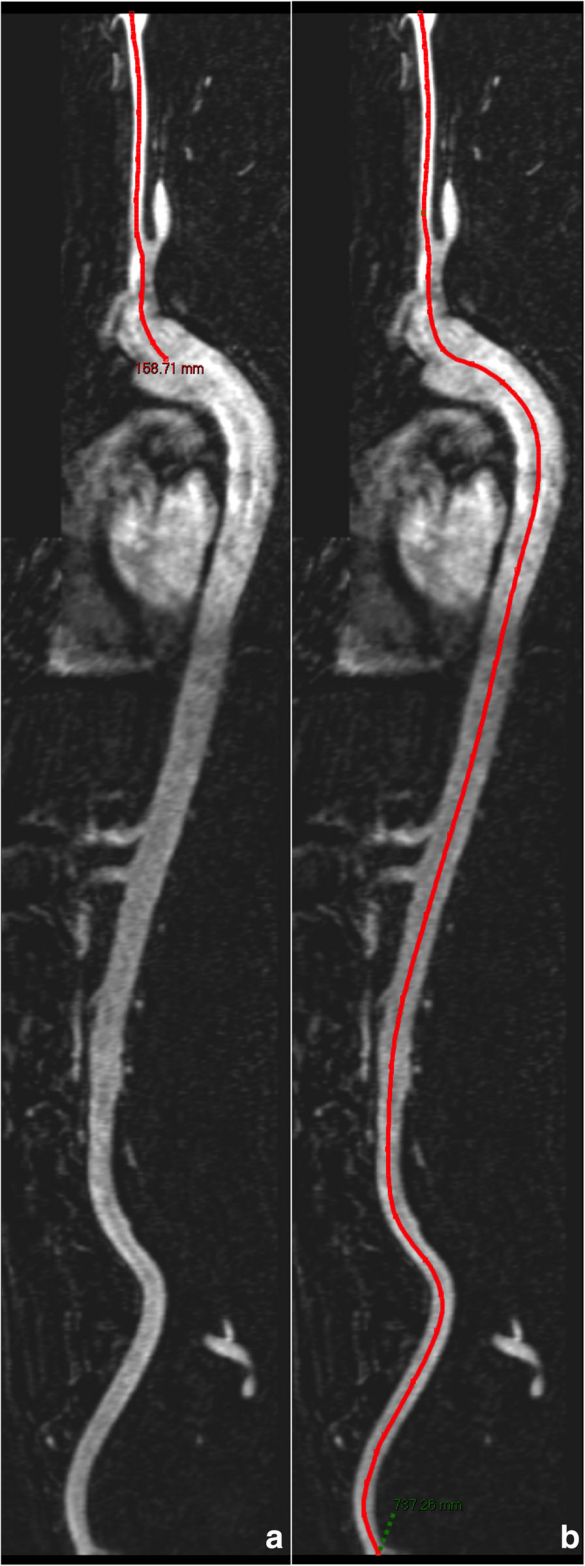
Curved multiplanar reformat of the arterial centerline from the bifurcation of the common carotid artery to the bifurcation of the common femoral artery with A- Right common carotid to aorta, and B- Right common carotid to common femoral artery, distances measured

The 3D WB-MRA datasets were viewed offline (Carestream PACS Client Suite Version 10.1 sp1, Rochester, NY, USA). An arterial centreline was drawn between the bifurcation of the right common carotid artery and the right common femoral artery. From this a curved multiplanar reformat of the vessel was generated. From this the distance from the carotid bifurcation to the centre of the aortic arch was measured (proximal distance) (See Fig. 1). The distance from the common carotid to the common femoral artery was measured, from which the aortic arch to the common femoral artery bifurcation distance (distal distance) was calculated as the carotid to arch distance subtracted from the total distance, with these representing the measures taken using the external tape measure technique. The distances were measured twice and an average of the two used for subsequent analysis. This distance was then used to recalculate the cf.-PWV using MRA measured distance (distal – proximal distance) divided by the carotid-femoral time interval produced by the SphygmoCor device. For the remainder of the article, the cf.-PWV calculated using the MRA derived arterial distance shall be referred to as cf.-PWV_MRA_.

#### Cardiac magnetic resonance (CMR) protocols

Cardiac magnetic resonance (CMR) imaging utilised a spine matrix and six-element body array matrix RF coils. TurboFLASH two-chamber, four-chamber and short axis localiser, and two-chamber and four-chamber cine images were acquired. Left ventricular assessment involved the acquisition of a horizontal long axis, vertical long axis and stacked short axis cine images using a retrospectively gated TrueFISP sequence (repetition time TR = 3.4 ms; echo time TE = 1.48 ms; flip angle = 50–60°; pixel area = 1.4 mm by 1.9 mm; slice thickness = 6 mm; inter-slice gap = 4 mm). Left ventricular mass (LVM), end diastolic volume (EDV), end systolic volume (ESV), stroke volume (SV) and ejection fraction (EF) were calculated from the short axis stack.

### MRI pulse wave velocity measurement

A retrospective ECG-gated gradient-echo pulse sequence with velocity encoding was applied to measure the through plane flow at two predefined locations in the ascending and abdominal aorta. The first slice was positioned axial through the aortic arch at the level of the pulmonary bifurcation, and the second slice was placed axial through the descending aorta immediately proximal to the renal arteries.

Imaging parameters included the following: echo time of 4.83 ms, repetition time of 14 ms, flip angle 20°, slice thickness of 8 mm, field of view at 350 mm, matrix size 256 × 256. The temporal resolution was optimised to ensure that 128 phases per cardiac cycle were obtained and a VENC of 150 cm/s. To determine the distance between the two aortic slices, a 2D gradient echo FLASH (fast low angle shot) was acquired of the aorta in a ‘candy stick’ double-oblique orientation. TR/TE 40/1.2 ms; flip angle 15°, slice thickness of 8 mm, 23 cardiac phases, 1 averages, a pixel size of 1.5 × 1.5 mm^2^, bandwidth of 475 Hz/ pix and breath hold scan time of average 9 s. Aortic PWV was calculated from the MRI images using the transit time method [4]. Image analysis was performed by a single observer using Segment version 1.9 R4339 (http://segment.heiberg.se) [13], blinded to the clinical status of the subjects. The up-slope of the arriving pulse wave at each location was calculated from the flow curves. The distance was measured along the aorta between the two analyses planes using candy stick FLASH and the time delay calculated as the time delay between the arrival of the foot of the pulse wave at the ascending aorta and abdominal aorta.

### Statistical methods

Descriptive statistics were used for the analysis of the demographic and clinical features of the cohorts with data expressed as mean ± standard deviation (SD) for continuously distributed variables, and n(%) for nominal data.. Normality of distribution was tested using Shapiro Wilk test; if the test failed, where possible standard transformations such as square root, reciprocal or logarithmic transforms were used to generate a Gaussian distribution. To test the null hypothesis that samples originate from the same population, a paired sample t-test was used for comparison of distance measurements and between technique PWV measurements. Spearman correlation coefficient and Bland-Altman plots were performed to further examine the differences between carotid-femoral and MRI PWV. One-way analysis of variance (ANOVA) and Kruskal–Wallis ANOVA by ranks was used for the non-parametric data to compare demographic data and PWV measurements between the four groups. Backward stepwise linear regression was used to determine the contribution of body metrics and contours to discordances between tape measure and MRI measured arterial pathlengths. All data were analysed using SPSS statistical package (version 21.0, SPSS Inc. Chicago, Illinois). Significance was assumed when *p* < 0.05.

## Results

One hundred fourteen study participants completed the study protocol undergoing cf.-PWV, whole body MRA and cardiac MRI. Of these 90 also underwent MRI PWV measurement using phase contrast MRI of whom 88 had analysable data, and thus used in the final analysis. Demographics and CMR measurements of the 4 groups are described in Table 1.

**Table 1:** Population demographics of the participants

Volunteer Demographics	Group 1 CVD+ DM+	Group 2 CVD- DM+	Group 3 CVD+ DM-	Group 4 CVD- DM-	*P* Value
N	23	41	25	25	
Age (years)	64 ± 7	62 ± 9	68 ± 9**	62 ± 8	0.18
Male	18(78)	24 (59)	17 (68)	10 (40)	0.05
Height (m)	1.71 ± 0.09	1.68 ± 0.09	1.66 ± 0.08	1.67 ± 0.10	0.33
Waist (cm)	106 ± 11^	104 ± 11^	98 ± 12	94 ± 14	<0.001
Hip (cm)	108.9 ± 8.5^	109.8 ± 9.3^	103.8 ± 8.7	104.2 ± 8.3	0.006
BMI (kg/m^2^)	30 ± 4	30 ± 5	29 ± 3	28 ± 4	0.27
Current/ex smoker	16 (70)	21 (51)	14 (56)	13 (52)	0.43
Hypertension	17 (74)*	23 (56)*	21 (84)**	7 (28)	<0.001
Systolic BP (mmHg)	135 ± 12	135 ± 13	137 ± 17	135 ± 15	0.96
Diastolic BP (mmHg)	73 ± 8	77 ± 7	76 ± 8	78 ± 9	0.14
Total cholesterol (mmmo/L)	3.85 ± 0.89*	3.92 ± 0.78*	4.08 ± 0.67*	5.07 ± 1.0*	<0.001
LDL Cholesterol (mmol/L)	1.71 ± 0.51*	1.97 ± 0.74*	2.07 ± 0.66*	2.69 ± 0.79	<0.001
HDL Cholesterol (mmol/L)	1.14 ± 0.33*	1.21 ± 0.29*	1.29 ± 0.45*	1.53 ± 0.44	<0.001
Triglycerides (mmol/L)	2.11 ± 1.01	1.64 ± 0.72	1.55 ± 0.79	1.86 ± 1.07	0.06
Creatinine (mg/mL)	81 ± 21	73 ± 19	82 ± 19	71 ± 16	0.007
HbA1c (mmol/mol)	7.6 ± 1.3^	7.3 ± 1.3^	5.5 ± 0.2	5.7 ± 0.2	<0.001
Medications					
Antihypertensive	19 (83)**	30 (73)*	19 (76)**	7 (28)	<0.001
Statin	18 (78)*	23 (56)*	23 (92)**	8 (33)	<0.001
Prior cardiovascular event^a^					
CAD	18 (81)		17 (74)		
Cerebrovascular	3 (14)		4 (17)		
LEAD	2 (9)		6 (26)		
Cardiac MRI					
LVM (g/m^2^)	61 ± 9**	55 ± 9	59 ± 9*	53 ± 9	<0.001
LVEDV (ml/m^2^)	71 ± 10	67 ± 12	73 ± 12	68 ± 10	0.14
LVESV (ml/m^2^)	25 ± 11	23 ± 8	26 ± 11	23 ± 7	0.49
LVEF (%)	65 ± 11	65 ± 8	65 ± 10	67 ± 7	0.95

Values expressed as Mean ± SD, or N (%)

BMI = body mass index; BP = blood pressure; CAD = coronary artery disease; LEAD = Lower extremity arterial disease. LVM = left ventricular mass; LVEDV = left ventricular end diastolic volume; LVESV = left ventricular end systolic volume; LVEF = left ventricular ejection fraction.

*p < 0.05 compared to group 4

**p < 0.05 compared to groups 2 and 4

^ *p* < 0.05 compared to groups 3 and 4

‘*p* < 0.05 compared to group 2

^**a**^groups add up to >100% as several individuals had more than one prior cardiovascular event

PWV distance measurements were significantly lower using MRA arterial centrelines than on external tape measure (tape measure distance = 537 ± 48 mm vs. MRA distance = 452 ± 33 mm, mean diff = 85.4 ± 54.0 mm, *p* < 0.001). This is predominantly due to an underestimation of the carotid arteries to the arch distance measurement, and an over-estimation of the arch to femoral artery measurement (See Table 2). As a result, cf.-PWV_EXT_ was significantly higher than the recalculated cf.-PWV_MRA_ (cf-PWV_EXT_ = 10.98 ± 2.63 ms^−1^ vs. cf.-PWV_MRA_ = 9.2 ± 2.04 ms^−1^, *p* < 0.001). Despite this the carotid femoral PWV calculated using the tape measure and using the MRA centerline showed a high degree of correlation (rho = 0.90, *p* < 0.001). A backward stepwise linear regression model using the difference between external tape measure differences and arterial centerline distances as the dependent variable and age, sex, height, weight, waist circumference, and hip circumference as independent variables was performed to better understand the source of the differences between the two measurement techniques. Age (B = −0.75 ± 0.45, *p* = 0.1), height (B = −187 ± 50.3, *p* < 0.001), waist (B = 1.29 ± 0.5, *p* = 0.011), and hip circumference (B = 2.17 ± 0.62, *p* = 0.001) were all significantly related to distance measurement discrepancy and combined accounted for 42.9% of the difference between the techniques.

**Table 2:** Comparison of the proximal and distal measurements using an external tape measure technique and arterial centerline technique

Distance measurement	External (A)	MRA (B)	*p*-value (A - B)
Carotid-Arch (mm)	92 ± 15	160 ± 17	<0.001
Arch-Femoral (mm)	629 ± 45	611 ± 39	<0.001
Distance for PWV calculation (mm)	537 ± 48	451 ± 33	<0.001

**Fig. 2:**
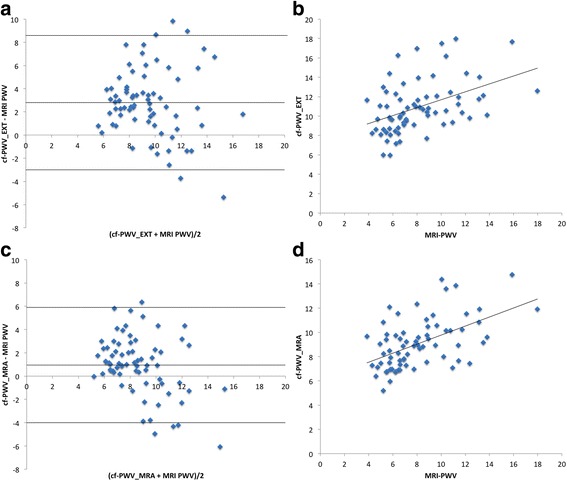
Scatter plot and Bland-Altman plots of cf.-PWV_EXT_ compared to MRI-PWV (A and B) and cf.-PWV_MRA_ with MRI-PWV (C and D)

MRI-PWV showed moderate correlation with cf.-PWV_EXT_ (rho = 0.49, *p* < 0.001) but showed a consistent bias with a significantly lower MRI-PWV than cf.-PWV_EXT_ (MRI-PWV = 8.12 ± 2.9 ms^−1^ vs. cf.-PWV = 10.92 ± 2.68 ms^−1^, *p* < 0.001). When cf.-PWV was recalculated using the inter-arterial distance from whole body MRA, correlation between the two techniques improved (*R* = 0.55, *p* < 0.001), and a significant reduction in the difference between the two techniques (MRI-PWV = 8.12 ± 2.92 ms^−1^ vs. cf.-PWV_MRA_ 9.08 ± 2.06 ms^−1^. Despite this improved correlation, and a significant reduction in the bias and a reduction in the inter-technique variability, as demonstrated in the Bland Altman plots in Fig. 2, the differences between the techniques remained significantly different (mean difference = 0.96 (95%CI 0.36–1.55 ms^−1^). Recalculation of the PWV using the MRA measurements improved the correlation between PWV and age (cf-PWV_EXT_ rho = 0.5, cf.-PWV_MRA_ rho = 0.65, *p* < 0.001 for both) and pulse pressure (cf-PWV_EXT_ rho = 0.5, cf.-PWV_MRA_ rho = 0.52, *p* < 0.001 for both), see Table 3 for a full list of correlates.

Neither cf.-PWV_EXT_ nor cf.-PWV_MRA_ differentiated between the four groups although the MRI PWV showed a trend towards differentiation between the groups (see Table 4). When the groups were combined into having CVD or no-CVD, a continued lack of difference between these groups was observed for both cf.-PWV_EXT_ (*p* = 0.68) and cf.-PWV_MRA_ (*p* = 0.39), however a significant difference was seen in MRI-PWV (CVD + ve MRI-PWV = 9.22 ± 3.37 vs CVD-‘ve MRI-PWV 7.22 ± 2.12 ms^−1^, *p* = 0.01).

**Table 3:** Comparison of the effects of recalculation of PWV on correlation with cardiovascular risk factors

	cf-PWV_EXT_		cf-PWV_MRA_		MRI-PWV	
	Rho	p	Rho	p	Rho	p
Age	0.50	<0.001	0.65	<0.001	0.49	<0.001
Systolic BP	0.50	<0.001	0.52	<0.001	0.44	<0.001
Diastolic BP	0.08	0.4	0.06	0.5	0.08	0.5
Pulse pressure	0.46	<0.001	0.52	<0.001	0.24	0.03
BMI	0.08	0.4	−0.15	0.4	-0.17	0.1
Smoking packyears	0.12	0.2	0.15	0.1	0.03	0.8
Total cholesterol (mmmo/L)	−0.09	0.3	−0.03	0.8	-0.04	0.7
LDL Cholesterol (mmol/L)	−0.1	0.3	−0.06	0.6	-0.03	0.8
HDL Cholesterol (mmol/L)	−0.12	0.2	−0.03	0.7	-0.1	0.4
Triglycerides (mmol/L)	0.12	0.3	0.06	0.5	0.1	0.4
Creatinine (mg/mL)	0.12	0.2	0.15	0.1	0.19	0.1
HbA1c (mmol/mol)	0.22	0.1	0.06	0.6	0.03	0.8
LVM	0.14	0.1	0.03	0.8	0.04	0.7

*BMI* body mass index; *BP* blood pressure; *CAD* coronary artery disease; *LEAD* Lower extremity arterial disease. *LVM* left ventricular mass

**Table 4:** Comparison of PWV between the four groups calculated using tonometry with an external tape measure technique or an arterial centerline technique for the distance measurement, and separately using MRI

PWV technique	Group 1 CVD+ DM+	Group 2 CVD- DM+	Group 3 CVD+ DM-	Group 4 CVD- DM-	p
cf-PWV_EXT_ (ms^−1^)	11.3 ± 3.0	11.2 ± 2.7	11.1 ± 2.4	10.2 ± 2.4	0.25
cf-PWV_MRA_ (ms^−1^)	9.2 ± 2.3	9.2 ± 1.9	9.7 ± 2.1	8.8 ± 1.8	0.5
MRI-PWV (ms^−1^)	9.2 ± 2.3	7.6 ± 2.4	9.2 ± 3.6	6.7 ± 1.6	0.09

Cf = carotid-femoral; MRA = distance measured using arterial centrelines obtained on whole body magnetic resonance angiography; MRI = Magnetic resonance imaging; PWV = Pulse wave velocity; EXT = distance measured using an external tape measure technique

## Discussion

In the current study we have shown a significant difference between the external distance measurement used for the calculation of PWV and the actual arterial pathlength. When this distance discrepancy is corrected for using intra-arterial distances measured on MRI there is a significant improvement in the agreement between PWV measured using tonometry or MRI.

The continued difference of approximately 1 ms^−1^ between the two techniques after accounting for this difference in distance measurements suggests part of the differences in the PWV is due to differences in the underlying arterial stiffness between the underlying vessels. Previous work has shown the PWV to increase throughout the aorta, although even in the distal aorta where the PWV was the highest, this was still 1 ms^−1^ lower than cfPWV [6]. Thus differences in the two measurements are almost certainly secondary to differences in carotid and iliac stiffness. Of these the iliac stiffness is the most likely to contribute to this difference as the carotid artery demonstrates a similar distensibility to the abdominal aorta, while the femoral arteries are significantly stiffer than both [14, 15]. The distorting effect of the iliac stiffness on this measurement has clinically significant implications, as even the corrected cfPWV did not differentiate between those with and without cardiovascular disease, whereas the aortic stiffness as measured on MRI was able to detect a significant difference in the PWV between these.

Our difference between cf.-PWV and MRI is consistent with prior studies, although is even more pronounced at 2.8 ms^−1^ than previous reports of 1.8 ms^−1^ [5, 6]. The most likely explanatory factor for this is the much higher BMI of the recruited participants in the current study compared with these two previous studies. Our findings of an 85 mm discordance between the external measurement technique and the actual arterial pathlength taken by the pulse wave is in agreement with prior work by Huybrechts et al. who found a difference in distance measurement of 81.8 mm between an arterial centreline and external tape measure distance [8]. This previous study found a significant confounding effect from age on pathlength measurement with only 4% of the discrepancy being accounted for by BMI. This is in contradistinction to our study where weight and hip circumference accounted for 42% of the measurement differences with only 1% of differences explained by age. These differences in findings are likely due to the much narrower age range combined with the more obese nature of our study participants compared to the wide age range and relatively normal weight in the previous study, as obesity is known to result in increased tape measure distances [16, 17]. However as those who are most likely to be assessed by PWV for risk assessment are likely to be older and overweight, rather than young and slim, our findings are likely to be more clinically relevant, and add weight to the proposal that direct caliper measured distance between the carotid artery and femoral artery should be the measurement technique of choice as these are least affected by body contours [7]. Creating a consistent technique for the accurate calculation of travelled pulse wave distance is crucial to the interpretation of these studies, and also to allow comparison between different techniques. We have shown that differences in PWV between MRI and tonometric measures can be largely accounted for by inaccuracies in these distance measurement techniques. Thus creation of suitable techniques may allow not just accurate PWV measurements but improved cross centre reproducibility and the ability to combine data from multiple techniques in systematic reviews and meta-analysis adding greater power to these. It may be possible to generate these measurements automatically from simple patient allometric measures such as sex, age, and height, and indeed several groups have tried exactly this [18, 19]. However one of these used the tape measure as the gold standard while the other used the aortic root to aortic bifurcation measurement, neither of which reflect the true path of the pulse wave as measured by carotid-femoral PWV. One option may be to use large whole body imaging studies such as the TASCFORCE and SHIP studies that would allow for the generation of an accurate aortic pathlength model based on thousands of individuals [20–22]. While this would require substantial effort and external validation, the benefits may be justified in terms of improved inter-centre and inter-individual reproducibility due to the removal of an extra step in the process of PWV and its incumbent potential for human error.

In our study we observed no significant difference between the groups using either cf.-PWV or MRI PWV. This is likely due to the relatively small sample size of our study, as using the same carotid-femoral technique in a larger population, of which the current group is a sub-study of, significant differences were observed between all groups [11]. Our lack of correlation between PWV and left ventricular mass is interesting as it has previously been posited that the importance of arterial stiffening is due to its effects on the afterload of the ventricle resulting in an increased work load with resultant adverse remodelling [23]. Previous studies demonstrating a correlation between the two have been performed in healthy volunteers or those with cardiovascular risk factors but no overt clinical cardiovascular disease [24, 25]. Thus PWV may have a greater impact on left ventricular mass earlier in the arteriosclerotic disease process with later left ventricular remodelling being affected by other processes such as coronary atherosclerosis, however longitudinal studies will be required to further elucidate this relationship.

There are several limitations within our current study. We used a three point technique for measuring the PWV distance externally, while current ESC guidelines would advocate the use of a direct carotid-femoral distance measurement with a correction factor of 0.8 to account for the shorter internal pathlength of the pulse wave and the simplicity of this technique [3]. However this is largely based on a single MRI study of 98 individuals, and other studies have suggested the currently used technique provides superior correlation with invasive aortic PWV over a direct distance measurement technique [18]. Our cf.-PWV and MRI scans were performed on different days, which is known to affect PWV measurement due to differing haemodynamic states [26], and may account for the lower correlation between the two techniques than has been seen in previous studies, but this does not change the fact that recalculation of PWV using an accurate distance measurement improves agreement between techniques. Finally, the MRI PWV and cf.-PWV use different techniques for determining wave arrival time which is known to affect the measurement and reproducibility, [27] however each technique was optimised to maximise its own intrinsic accuracy and reproducibility.

In conclusion, differences in PWV measurement between carotid-femoral PWV and MRI measured central aortic PWV can be predominantly explained by inaccuracies introduced by the use of simple surface measurements to represent the convoluted arterial path between the carotid and femoral arteries. Correction for this may in future allow more direct comparison between the techniques thus strengthening systematic reviews and meta-analyses.
